# Evaluation of the Effectiveness of a Nursing/Physiotherapy Program in Chronic Patients

**DOI:** 10.3390/ijerph16122236

**Published:** 2019-06-25

**Authors:** Remedios López-Liria, Francisco Antonio Vega-Ramírez, José Manuel Aguilar-Parra, David Padilla-Góngora, Rubén Trigueros-Ramos, Patricia Rocamora-Pérez

**Affiliations:** 1 Health Research Centre, Department of Nursing, Physiotherapy and Medicine, University of Almería, Carretera del Sacramento s/n, La Cañada de San Urbano, 04120 Almería, Spain; 2 Distrito Sanitario Poniente, Jesús de Perceval, 22. El Ejido, 04700 Almería, Spain; franavega@hotmail.com; 3 Department of Psychology, University of Almería, Carretera del Sacramento s/n, La Cañada de San Urbano, 04120 Almería, Spain; dpadilla@ual.es (D.P.-G.); ruv_1987@hotmail.com (R.T.-R.)

**Keywords:** home health care, physiotherapy, case management, functional independence, chronic condition, multiple pathology

## Abstract

This study aimed to evaluate the functional impact of a shared intervention model by the mobile physiotherapy and rehabilitation team (MPRT) and primary care case management nurses (PCCMNs) on chronic patients. This was a prospective, observational study involving 1086 patients (mean age, 80 years; 63.7% females) in the province of Almeria, which was conducted between 2004 and 2018. Most of the registered diseases included cerebrovascular and neurological diseases (56.7%), osteoarticular diseases (45.3%), diabetes mellitus (25.7%), cardiovascular diseases (25.5%), and chronic respiratory diseases. The study included a home care intervention by the MPRT and PCCMNs and included the following main outcome measures: age, sex, main caregiver, disabling process (ICD-9), type and number of inclusion categories for chronic disease, initial and final Barthel index (BI), treatment or intervention on the patient (techniques), objectives, and number of sessions. The main techniques used were kinesiotherapy (44.6%) and caregiver training (23%), along with technical aid. An equation predicting the patients’ final BI, according to the initial BI, was constructed using multiple linear regression modelling. A marked improvement in functional capacity was found after an average of 10 physiotherapy sessions. A lower patient age was correlated with a higher functional capacity, both initial and final BI, as well as a greater number of sessions.

## 1. Introduction

Currently, one of the main goals of health service providers is to enable patient-centered care in their own environment. This is achieved by ensuring cooperative and coordinated services among different care providers, including the patient’s family and social and community resources [1]. 

In Andalusia (Spain), mobile physiotherapy and rehabilitation teams (MPRTs) were established in primary care (PC) in 2002 and included in the regional Family Support Plan with the aim of improving patient and caregiver access to these services in the home environment [2]. Concurrently, the development of advanced practice nursing roles became a reality that spread internationally [3], although implementation of the roles and nursing competences have varied according to professional practices in each country [4]. In Andalusia, within the aforementioned regional Family Support Plan, the figure of the primary care case management nurse (PCCMN) was established with a unified service catalogue of both social and healthcare resources. This was done in order to improve the coordination between specialized care (SC) and PC, increase patient satisfaction, and provide high-quality, cost-effective healthcare solutions [2,5]. 

Chronic diseases are long-term, slow-developing conditions with serious adverse side effects that affect one’s quality of life and have important economic repercussions, causing a high degree of disability and dependence [6]. They represent a challenge to both the healthcare system and patients’ families, and are often an important source of stress, which can have a serious impact on the caregiver [1,7,8]. 

Chronic diseases are one of the foremost challenges to healthcare, owing to their high prevalence and morbidity. Although, owing to medical progress, the aging population experiences an increased survival rate, many chronic patients turn to emergency services, develop comorbidities and complications, and require complex treatments and hospitalization [9]. In Spain, 75% of healthcare spending goes towards diseases such as diabetes, arthritis, chronic respiratory diseases, obesity, and chronic mental health disorders, owing to the complex nature of chronic morbidities [10,11,12]. 

The increased demand for home care (HC) for chronic patients may be explained by the fact that most elderly people and dependents prefer to be treated at home [13]. Predictive factors for HC include advanced age, cognitive impairment, incapacitating chronic disease, early hospital discharge, and lack of autonomy to carry out the basic activities of daily living [14,15,16,17].

For these reasons, strategic guidelines should be established to achieve a fully developed integral HC system for chronic patients that guarantees the best results in terms of overall health and quality of life, delivery of improved services, cost-effective interventions, greater cohesion of health care teams, a sustainable healthcare system, and high patient satisfaction levels [18].

According to a previous study [14,15,16], we began with the hypothesis that HC interventions by the MPRT and PCCMNs contribute to a greater functional recovery of chronic patients in PC. The objective of this study was to evaluate the functional impact of a shared intervention model by the MPRT and PCCMNs, in a sample of 1086 chronic patients.

## 2. Materials and Methods

We performed a prospective observational study to analyze the frequency, determinants, and factors related to different diseases in PC chronic patients in the province of Almeria. The clinical history of 4762 patients, registered between 2004 and 2018, were analyzed, and 1086 chronic patients were included who met the following criteria: patients referred by PCCMNs to the MPRT who had received care according to the service action protocol [19], suitability (health care assistance process included in the home care processes catalogue), accessibility (inability to attend a medical center owing to architectural barriers), and safety (travel represents a risk due to the patient’s health) or comorbidity. Furthermore, patients had to fulfil at least one of the chronic patient criteria as defined by the Andalusian Government’s Integrated Health Care Assistance Process for Multiple Pathology [20,21], such as ‘present one or more of the chronic symptomatic and progressive diseases of the eight defined’ (Table 1). We used disabling process (ICD-9) and chronic patient criteria according to the definition of the Andalusian Government’s Integrated Health Care Assistance Process for Multiple Pathology [20,21] as a measure of classification by pathology, because they adhere to the guidelines of Andalusian Public Health and allow multiple inferences or comparisons between centers. The primary care case management nurse may receive patients referred from any SC service, or other PC professionals, and be asked for an assessment, management, and/or intervention process. They support PC and coordinate SC services, including coordinating care for patients returning home after discharge. PCCMNs reduce the impact of the immobility (adaptation or technical aids) and caregiver overload by supporting relatives (to help them take care of their own physical and mental health) and providing access to other health services such as the MPRT.

Patients were excluded if they refused to receive physiotherapy, or if it was contraindicated. The study variables were as follows: age, sex, main caregiver identity, disabling process (ICD-9), type, and number of inclusion categories for chronic disease (Table 1), and the initial and final Barthel index (BI) to determine the degree of patient dependence to carry out basic activities of daily living. Depending on the score, the patients were classified as total dependence (0–20), severe (21–60), moderate (61–90), little dependence (91–99), and independence (100) [22]. Rehabilitation treatment or intervention of the patient (techniques such as functional exercises, electrotherapy, caregiver training, or how use technical aids), and objectives and number of sessions were also included among the variables.

### Procedure and Data Analysis

Prior to commencing the study, permission was sought from the pertinent ethics and sciences committee of the province of Almeria (CEIC-AL 39/2012). Data were obtained in strict adherence to current privacy and data protection laws, and guidelines from the International Committee of Medical Journal Editors. All patients provided written informed consent before treatment, in accordance with the Helsinki Declaration.

Descriptive and bivariate analyses were carried out using SPSS version 23 (IBM, Armonk, NY, USA); analyses sought possible associations between dependent and independent variables. *t*-tests for related samples were performed to calculate the changes between scores for the initial and final BI in each clinical category. *t*-tests for independent samples were used to calculate differences in functionality (BI) through sex. We performed an analysis of variance (ANOVA) test for the initial and final BI values, and the patients’ age groups (60–69, 70–79, 80–89, and 90–99). If all criteria were met (normality and homogeneity of the variance with the Levene test), multiple comparisons were made using the Bonferroni test; when homogeneity was not fulfilled, a Dunnett’s T3 test was performed.

A multivariate analysis (MANCOVA) was conducted to assess the differences between clinical categories, with sex and age (independent variables) and initial BI as covariates; this analysis was complemented by the calculation of effect size by η_p_^2^.

Additionally, relationships between quantitative variables (age, number of sessions, and initial and final BI) were assessed through correlation measurements (Pearson linear), and the multiple regression was obtained as determined by the predictive model in relation to the BI values of the patients.

## 3. Results

The average age of the 1086 patients was 80.42 years (SD = 12.3), of which 63.7% were women. The reasons for referral to the unit were as follows: multimorbidity, advanced age (85.2%), and architectural barriers in the home (14.8%).

The main caregiver in the home was identified primarily as the daughter (30.1%), followed by the family (17.9%); wife (12.5%); others (7.3%); son (6.5%); husband (5.1%); children (4.9%); and, finally, grandchildren (0.8%). Home assistants collaborated in 11.7% of cases, while 3.3% of elderly people had no caregiver.

The most common disabling processes were as follows: consequences of immobility (25.7%); motor impairment (19.2%), cerebrovascular disease (12.3%), Alzheimer’s disease (10.8%), fractured hip (5.3%), chronic obstructive pulmonary disease (COPD, 3.7%), and Parkinson’s disease (3.2%); the remainder represented less than 2% of the total.

The main objectives of treatment were to improve functioning (27.5%), caregiver training (14.9%), walk training (15.1%), and pain relief (2.5%), and to increase respiratory capacity (3.9%). 

The different rehabilitation techniques applied were grouped under five categories as follows: functional exercises (44.6%), functional exercises and electrotherapy (5.8%), caregiver training (23%), functional exercises and caregiver training (17.3%), and technical aids (9.3%). The average number of physiotherapy sessions was 9.8 (SD = 10.2).

The difference between the average initial BI ($\bar{X}$ = 33.4; SD = 27.0) and final BI ($\bar{X}$ = 44.7; SD = 33.5) was statistically significant; student’s t-test was performed for related samples, in the sample of 1000 patients (*t* = −20.47; confidence interval, CI 95%: −12.32 to −10.16; and *p* < 0.001). Table 2 indicates the values of the initial and final BI according to the patient’s disabling process (clinical category).

Analysis of the inclusion criteria for the patients into single or multiple chronic pathologies revealed that 31.5% of the sample (*n* = 326) had one inclusion category, 28.2% had two categories (*n* = 292), 21.3% (*n* = 220) had three, 8.8% (*n* = 91) had four, 3.4% (*n* = 35) had five, and 0.3% (*n* = 3) had six. The most prevalent category for referral was E (56.7%; *n* = 584), stroke, and neurological and cognitive impairment, followed by H (45.3%; *n* = 467) chronic osteoarticular disease; F, 25.7% (*n* = 265), peripheral artery disease (PAD), and diabetes mellitus with retinopathy; A, 25.5% (*n* = 263), heart failure; C, 17,6% (*n* = 181), chronic respiratory disease; G, 14.3% (*n* = 147), chronic anemia and solid or hematologic neoplasm; B, 13.3% (*n* = 137), autoimmune disease and renal disease; and D, 7.9% (*n* = 81), inflammatory bowel disease (IBD) and chronic liver disease.

The quantitative variables (age, initial BI, final BI, and number of sessions) were analyzed to determine the existence of any sex-related differences (Table 3). No statistically significant differences were found in the number of physiotherapy sessions performed for men and women (*t* = 0.27; CI 95%: −1.25 to −1.28; and *p* = 0.979).

On analyzing the statistically significant differences between age groups (60–69, 70–79, 80–89, and 90–99 years) relative to the initial and final BIs, statistically significant differences were found in some groups (*p* < 0.001). These age groups also fulfilled the applicability hypothesis; in the case of the initial BI, all the conditions required to carry out an ANOVA test were fulfilled, indicating a statistically significant difference between groups (F = 5.538; *p* = 0.001); analysis of post hoc Bonferroni data (difference between means I–J = 9.84; *p* = 0.049) showed differences between the 60–69 years ($\bar{X}$ = 37.77) and the 90–99 years age group ($\bar{X}$ = 27.93). Statistically significant differences (I−J = 10.43; *p* = 0.001) were also found between the 70–79 ($\bar{X}$ = 38.37) and the 90−99 years age group ($\bar{X}$ = 27.93).

Regarding the final BI, the ANOVA analysis revealed statistically significant differences between the groups (F = 7.640; *p* = 0.000). The post hoc Bonferroni test (I-J= 16.06; *p* = 0.000) revealed differences between the 70–79 years group ($\bar{X}$ = 51.67) and the 90–99 years group ($\bar{X}$ = 35.60). Statistically significant differences were also found (I−J = 10.04; *p* = 0.004) between the 80–89 years group ($\bar{X}$ = 45.65) and the 90–99 years age group ($\bar{X}$ = 35.60).

A MANCOVA inferential analysis concluded that there were statistically significant differences between the initial BI indices by clinical category (*p* < 0.001, F(8000) = 14,540, Wilks’ Lambda = 0.881; η^2^ = 0.119). There were statistically significant differences due to sex (*p* ≤ 0.001, F(8000) = 6505, Wilks’ Lambda = 0.943; η^2^ = 0.057] and age (*p* = 0.039, F(24,000) = 1568, Wilks´ Lambda = 0.957; η^2^ = 0.014). There were no significant differences due to sex × age (*p* = 0.387, F(24,000) = 1057, Wilks’ Lambda = 0.971; η^2^ = 0.010). Considering the effect size, these differences showed a low influence.

Table 4 shows that these differences in the initial BI of the participants were found in all categories except D, F, and G. Sex-related differences relative to the initial BI were only found in categories C, E, and H; age−related differences were found in categories A, F, and H.

Table 5 presents the Pearson correlation coefficient (PCC) for the quantitative variables (age, initial and final BI, and number of sessions), and shows that a higher patient age was correlated with a lower initial and final BI, and fewer treatment sessions (although the association was not very strong).

Finally, the following equation was used to predict the final BI according to the initial BI and the number of sessions, using the following multiple linear regression model:

Final Barthel index = −3.27 + 1.049 × Initial Barthel index + 0.70 × number of sessions + error(1)

The relationship between the three predictive variables and the final BI is strong because the correlation coefficient was 0.892 (saturated linear model); hence, 80% of the final BI variance was explained by the combination of predictive variables. As confirmed by the ANOVA, the regression was significant (F = 1227.46; *p* < 0.001). An inferential analysis on the saturated model parameters concluded that only the initial BI (β = 0.844 *p* < 0.001, *t*(941) = 57.024 and semipartial correlation = 0.839) and the number of sessions (β = 0.216 *p* < 0.001, *t*(941) = 14.555 and semipartial correlation = 0.214) were significant; however, age was not significant (β = 0.027 *p* = 0.066, *t*(941) = 1.841, and semipartial correlation = 0.027). 

## 4. Discussion

The World Health Organization [23] states that ischemic heart disease and stroke, COPD, and cancer account for 63% of all deaths. In medium and low-income countries, 80% of deaths are due to chronic diseases, in both men and women. The Spanish Institute of Statistics [24] has established an average of 2.8 chronic diseases per person in people aged 65–74 years, increasing to 3.23 in those aged ≥75 years [25]; approximately 50% of chronic patients fall into more than one category [12]. Chronic pathologies are the cause of 80% of healthcare assistance requests in PC, 60% of hospitalizations, and account for up to 70% of the national health expenditure [25]. In most studies, the average age of chronic patients with multiple pathologies is 66–82 years [26,27,28,29], coinciding with the results herein. The profile of the principle caregiver at home (daughter or wife in most cases) also coincides with the results of most studies in this field [30,31,32,33,34,35].

Some studies have indicated that diseases in A and F categories are the most frequent reasons for referrals (Table 1), corresponding to heart failure, ischemic cardiopathy, PAD, and diabetes with proliferative retinopathy or symptomatic neuropathy [27,29]. This differs from the results of the current study, which indicates category E (stroke and neurological conditions), followed by H (osteoarticular disease) as the most frequent. The National Health Survey [20,24] confirmed that at least one in six adults suffer from lumbar back pain (18.6%), arterial hypertension (18.5%), arthrosis, arthritis, or rheumatism (18.3%), high cholesterol (16.4%), or chronic cervicalgia (15.9%); the percentages of these chronic pathologies´ were dependent on the ages of the patients. These figures may explain the high percentage of osteoarticular pathologies (45.3%) found in our study, for which the average age was 80 years. 

This study evaluates the functional impact of a shared intervention model by the MPRT and PCCMNs, in a sample of 1086 chronic patients.

The objectives of treatment for geriatric patients depend on patient evaluation, their clinical stage, mental state, and adherence to treatment. The main objectives include improvement of quality of life, recovery, and normalization of sensory motor functions, prevention of complications during the course of the disease (particularly during confined to bed periods), optimization of the remaining physical and psychic faculties that are not affected, and recovery of those affected, maintaining functional independence and ambulation as long as possible [36]. In line with the above, the main objectives of treatment in the present study were to improve functionality (27.5%), walk training (15.1%), and caregiver training (14.9%).

The case management nurse´s activities were carried out on behalf of patients and their specific needs. These included procedures, assessments, providing physical care, and counselling; promoting innovative patient care and facilitating the optimal progression of patients through the healthcare system. They assessed patient/family response to therapy and modified the plan of care, performing interdisciplinary coordination in planning in order to optimize the patient’s health.

The rehabilitation techniques applied with the aim of achieving these objectives were, principally, functional exercises (approximately 50%), caregiver training (a third), functional exercises combined with caregiver training (17.3%), and functional exercises combined with electrotherapy (a small percentage). These results are in line with those obtained in previous studies [14], although the average number of sessions (9.8) was slightly lower (12.8) [14].

There was a difference of approximately 10 points between the initial and final BI, once interventions by PCCMNs and the MPRT were initiated. In a study by García Morillo et al. [37], 16% of cases progressed by more than 10 points in the BI, between baseline and final assessment (discharge). In our study, it is noticeable how this difference varies according to the clinical category (Table 2). Patients in category D (chronic liver disease) obtained the best functional results with a difference of 19 points, followed by category C (chronic respiratory disease), B (autoimmune and chronic renal diseases), and A (heart disease). The least functionality was obtained in categories G (chronic anemia and solid or hematologic neoplasm), E (stroke and neurological conditions), and H (osteoarticular diseases). The post-treatment results were not influenced by sex. 

In our study, a higher age correlated with a lower initial and final BI, and a lower number of treatment sessions; these results agree with those of a similar study on patients with motor impairment by Vega-Ramírez et al [38]. However, we found differences in the predictive models generated by both studies, which in our case allowed us to predict the final BI according to the initial BI and the number of sessions; as a result, we found that age was not significant, unlike the predictive model generated in the previous research [38]. 

Recently, the Cochrane Library reviewed the effectiveness of patient-centric interventions and health services designed to improve the condition of patients with multiple pathologies (those with two or more chronic diseases) in PC and the community [21]. The review consisted of 18 clinical trials, 9 of which focused on the definition of comorbidity conditions with an emphasis on depression, diabetes, and cardiovascular diseases. In 12 of these studies, the predominant intervention was case management or multidisciplinary teamwork [21]. In 2016, the Andalusian Health Service [9] confirmed, in agreement with our study, the need to develop a personalized assistance plan for each complex chronic patient, through a “multiprofessional evaluation and action plan” to be carried out in a coordinated manner and included specific objectives for the patient and team. 

The advent of new roles, such as the case management nurse, improvements in the care, attention to certain processes, and changes in the location of certain services (such as mobile physiotherapy and rehabilitation) are key to services that are adapted to the needs of the chronic patient [1]. The solution to the problem of chronicity must be offered from an integral viewpoint, with preventative interventions and coordination between the various implicated sectors, using shared guides and protocols, in addition to the participation of patients and their caregivers and/or families [1,39]. 

Several studies confirm the role of PCCMNs in facilitating the sustainability of services and the effectiveness of results obtained in chronic and complex-chronic patients [40]. The main contributions of PCCMNs to chronic patients have been classified into four principal areas in a study by Applevy and Camacho-Berajano: health or care management results, team-work coordination, quality of services, and patient interaction and relationship [18].

The effectiveness of PCCMNs in Andalusia is the subject of continued study, with the aim of making more improvements. In 2016, López-Liria et al [41] concluded that home-based rehabilitation for stroke patients was at least as effective as out-patient hospital care in terms of reducing side effects and patients regaining their functional independence. Prior to this, similar studies confirmed the effectiveness of treatments in patients with knee replacements [42]. Furthermore, Vega-Ramírez et al [38], in a study of 473 home-based patients with motor impairment or complications due to immobilization and prolonged bed rest, described marked improvements in terms of the functional capacity for basic activities of daily living with an overage of 10 physiotherapy sessions in PC, which agrees with our findings.

Descriptive studies such as these help create predictive models that allow for the stratification of the population in accordance with their healthcare needs, which enables health services to be proactive, arranging specific intervention according to their concrete needs [43].

This study has some limitations. The patients were not randomly chosen but arbitrarily or intentionally, which may affect the external validity. The selection was conditioned by the availability of resources in the geographic area of the mobile team’s operations. However, the exhaustive data collection and the way these were collected provided information on the needs of these patients and the resources available to them. Similarly, we also obtained information from the professionals involved, their shared interdisciplinary knowledge, and consensus on the best strategies, in order to offer improved quality care for this particularly vulnerable population. This information has enabled this service to be quantitatively and qualitatively described, including its benefits and the needs detected, so that approximate estimates can be made of the socio-health resources required, both in the service offered and in the patients’ homes, as well as to propose plans for improvement. With similar studies that measure the cost effectiveness of these treatments, we could save on economic resources dedicated to polymedication or dependence care (fewer hours of care or avoidance of unnecessary institutionalization), offering greater functional independence to people, and improving the quality of life of their families and caregivers. It is necessary to integrate these services in order to provide more effective and efficient care to patients with complex health problems, beyond individual approaches with disparate organizational alternatives, and pending to be conclusively evaluated.

## 5. Conclusions

This study examines the care of chronic patients. Patient-centered care in their environment presents a current and future socio-health challenge, including an increasingly prioritized healthcare trend. 

The home intervention model shared between the PCCMN and MPRT in PC allows for an improvement of functional capacity in these chronic patients, with an average of 10 physiotherapy sessions. A lower patient age correlates with higher initial and final functional capacity, as well as a greater number of sessions.

The findings of this study have enabled a quantitative and qualitative description of the integrated services provided to a chronic HC patient. The results also reveal the benefits and limitations of the combined services, which are undeniably useful to estimate the socio-health resources needed in the service offered and in the patients’ homes, as well as propose future plans for improvement. 

The introductions of new roles, such as the PCCMN, or changes in the location of certain services, such as the MPRT, are two examples of the adaptation of healthcare services to the needs of chronic patients.

It is necessary to improve the delivery of HC for chronic patients seeking superior cost-effective health interventions, quality of life, and satisfaction, as well as that of their caregivers and the care team, thereby contributing to the sustainability of the healthcare system.

## Figures and Tables

**Table 1:** Categories of patients with multiple pathologies.

Category A A.1. Heart failure NYHA1-grade II (symptoms with normal physical activity); A.2. Ischemic cardiomyopathy.
Category B B.1. Vasculitis and systemic autoimmune diseases; B.2. Chronic renal disease defined by elevated creatinine levels (>1.4 mg/dL for men and >1.3 mg/dL for women) or proteinuria 2, for 3 months.
Category C C.1. Chronic respiratory disease with dyspnea grade II (MRC3 scale, dyspnea after a few minutes of walking at own pace on the level) or FEV 1 < 65%, or SaO_2_ ≤ 90%.
Category D D.1. Chronic inflammatory bowel disease; D.2. Chronic liver disease with portal hypertension.
Category E E.1. Stroke; E.2. Neurological disease with permanent motor impairment and limitations on basic activities of daily living (Barthel Index < 60); E.3. Neurological disease with permanent (or at least moderate) cognitive impairment (five or more errors in Pfeiffer’s test: SPMSQ).
Category F: F.1. Symptomatic peripheral arterial disease (PAD); F.2. Diabetes mellitus with proliferative retinopathy or symptomatic neuropathy.
Category G: G.1. Chronic anemia due to gastrointestinal disorders or acquired hemopathy non-subsidiary to treatment with curative aim with Hb < 10 mg/dL in 2 quarterly tests; G.2. Solid or active hematological neoplasia non-subsidiary to treatment with curative aim.
Category H: H.1. Chronic osteoarticular disease causing limitations on basic activities of daily living (Barthel index < 60).

NYHA: New York Heart Association; MRC: Medical Research Council; FEV: Forced expiratory volume; SPMSQ: Short Portable Mental Status Questionaire.

**Table 2:** Student’s *t*-test for initial and final Barthel index (BI) per clinical category.

Categories	*n*	Initial BI $\bar{X}$ (SD)	Final BI $\bar{X}$ (SD)	CI 95%	T-Student	Significance	*d*
A	263	37.90 (26.93)	51.26 (32.80)	−15.78/−10.92	−10.828	*p* < 0.001	0.445
B	137	38.61 (26.34)	52.35 (31.94)	−17.23/−10.24	−7.784	*p* < 0.001	0.469
C	181	41.82 (27.53)	55.68 (32.37)	−16.65/−11.06	−9.785	*p* < 0.001	0.461
D	81	36.15 (26.56)	55.20 (32.52)	−23.70/−14.40	−8.163	*p* < 0.001	0.641
E	584	26.20 (23.64)	36.51 (31.03)	−11.73/−8.89	−14.257	*p* < 0.001	0.373
F	265	33.19 (23.91)	45.56 (31.04)	−14.61/−10.11	−10.841	*p* < 0.001	0.446
G	147	31.89 (26.58)	40.91 (32.54)	−12.22/−5.810	−5.565	*p* < 0.001	0.303
H	467	36.70 (27.59)	47.65 (32.81)	−12.58/−9.31	−13.148	*p* < 0.001	0.361

**Table 3:** Student’s *t*-test for independent samples (sex).

Variables	Women $\bar{X}$ (SD)	Men $\bar{X}$ (SD)	T-Student	*p*	95% CI	*d*
Age	81.40 (11.89)	78.70 (12.89)	3.487	0.001	1.18/4.22	0.217
Initial BI	33.18 (26.63)	33.82 (27.50)	−0.358	0.721	−4.13/2.85	0.023
Final BI	43.87 (33.87)	46.06 (33.08)	−0.974	0.330	−6.59/2.21	−0.065
Physiotherapy sessions	9.83 (10.47)	9.81 (9.81)	0.027	0.979	−1.25/1.28	0.001

**Table 4:** Multivariate analysis (MANCOVA) analysis performed to assess the influence of clinical categories, sex, age, and initial BI (Barthel index).

Variables	Categories	F	*p*	Etha Squared
**Initial BI**	**Category A**	10.860	0.001	0.012
	**Category B**	6.393	0.012	0.007
	**Category C**	22.048	0.000	0.025
	**Category D**	0.471	0.493	0.001
	**Category E**	90.365	0.000	0.095
	**Category F**	0.240	0.624	0.000
	**Category G**	0.809	0.369	0.001
	**Category H**	10.482	0.001	0.012
**Sex**	**Category A**	2.121	0.146	0.002
	**Category B**	0.179	0.672	0.000
	**Category C**	23.047	0.000	0.026
	**Category D**	0.132	0.717	0.000
	**Category E**	7.409	0.007	0.008
	**Category F**	0.015	0.903	0.000
	**Category G**	3.251	0.072	0.004
	**Category H**	10.365	0.001	0.012
**Age**	**Category A**	3.690	0.012	0.013
	**Category B**	1.209	0.305	0.004
	**Category C**	0.504	0.680	0.002
	**Category D**	0.526	0.664	0.002
	**Category E**	0.426	0.735	0.001
	**Category F**	2.681	0.046	0.009
	**Category G**	0.402	0.752	0.001
	**Category H**	4.361	0.005	0.015
**Sex × Age**	**Category A**	0.733	0.532	0.003
	**Category B**	0.754	0.520	0.003
	**Category C**	2.280	0.078	0.008
	**Category D**	0.965	0.408	0.003
	**Category E**	1.561	0.197	0.005
	**Category F**	1.733	0.159	0.006
	**Category G**	0.465	0.707	0.002
	**Category H**	0.488	0.691	0.002

**Table 5:** Correlations for age, number of sessions, and initial and final Barthel index.

Pearson Correlation Coefficient		Age	Number of Sessions	Initial BI	Final BI
Age	Pearson correlation coefficient (PCC)	1	−0.082 **	−0.022	−0.013
	Sig. (bilateral)		0.007	0.493	0.682
	*n*	1085	1084	982	961
Number of sessions	PCC	−0.082 **	1	0.108 **	0.296 **
	Sig. (bilateral)	0.007		0.001	0.000
	*n*	1084	1085	981	960
Initial BI	PCC	−0.022	0.108 **	1	0.867 **
	Sig. (bilateral)	0.493	0.001		0.000
	*n*	982	981	982	946
Final BI	PCC	−0.013	0.296 **	0.867 **	1
	Sig. (bilateral)	0.682	0.000	0.000	
	*n*	961	960	946	961

** Correlation is significant at 0.01 (bilateral).

## References

[1] Herrera J.C.M., Asencio J.M.M., Kaknani S., Mayor S.G. (2016). Situaciones de cronicidad compleja y coordinación sociosanitaria. Enferm. Clín..

[2] Servicio Andaluz de Salud (2002). Documento Plan de Atención a las Familias. Estrategias de Mejora para la Atención Rehabilitadora y Fisioterapéutica en Andalucía. Equipos Móviles.

[3] Schober M., Affara F.A. (2009). International Council of Nurses: Advanced Nursing Practice.

[4] Gardner G., Chang A., Duffield C., Doubrovsky A. (2013). Delineating the practice profile of advanced practice nursing: A cross-sectional survey using the modified strong model of advanced practice. J. Adv. Nurs..

[5] Garcés J., Ródenas F. (2015). La gestión de casos como metodología para la conexión de los sistemas sanitario y social en España. Atención Primaria.

[6] World Health Organization Informe Sobre la Situación Mundial de las Enfermedades no Transmisibles 2010. WHO Library Cataloguing-in-Publication Data. Global Status Report on Noncommunicable Diseases 2010. http://www.who.int/nmh/publications/ncd_report2010/es/.

[7] Delicado M.V., Delicado M. (2011). Las cuidadoras. Cuidadoras Familiares: Calidad de Vida, Repercusión de los Cuidados y Apoyos Recibidos.

[8] Pérez-Cruz M., Muñoz-Martínez M.A., Parra-Anguita L., del-Pino-Casado R. (2017). Coping and subjective burden in primary caregivers of dependent elderly relatives in Andalusia, Spain. Atención Primaria.

[9] Montero A., Vázquez A., Zayas J.J., Espejo P., Martínez A. (2016). Estrategia de Cuidados de Andalucía Plan Andaluz de Urgencias y Emergencias. Equipos Móviles de Cuidados Avanzados. Servicio Andaluz de Salud. http://www.juntadeandalucia.es/servicioandaluzdesalud/library/plantillas/externa.asp?pag=/contenidos/gestioncalidad/desainnovacion/desa_eqmovcuid.pdf.

[10] Bengoa R. (2007). Innovaciones en la gestión de las enfermedades crónicas. Jano Med. Humanid..

[11] Coderch J., Sánchez-Pérez I., Ibern P., Carreras M., Pérez-Berruezo X., Inoriza J.M. (2014). Predicción del riesgo individual de alto coste sanitario para la identificación de pacientes crónicos complejos. Gaceta Sanitaria.

[12] Sabaté M.Q., Ponsa M.C., Revuelta E.A., Requejo S.R., Rebull F.G., Peña E.B. (2014). Evaluación de un programa de atención a la cronicidad en Girona (CRONIGICAT). Atención Primaria.

[13] Montagut F., Flotats G., Lucas E. (2014). Rehabilitación Domiciliaria. Principios, Indicaciones y Programas Terapéuticos.

[14] López−Liria R., Padilla D., Catalán D., Arrebola C., Garrido P., Martínez M.C., Zurita F. (2010). Análisis de la actividad en las Unidades Móviles de rehabilitación−fisioterapia. Atención Primaria.

[15] López-Liria R., Padilla-Góngora D. (2011). Atención Integral en el Domicilio del Paciente con Accidente Cerebrovascular.

[16] López-Liria R., Padilla-Góngora D., Catalán-Matamoros D.J., Rocamora-Pérez P., Martínez-Cortés M.C., Rodríguez-Martín C.R. (2012). Análisis de las patologías con mayor prevalencia en las Unidades Móviles de Rehabilitación y Fisioterapia de la provincia de Almería. Gaceta Sanitaria.

[17] Martín−Zurro A., Jodar G. (2011). Atención Familiar y Comunitaria.

[18] Applevy C., Camacho-Bejarano R. (2014). Challenges and opportunities: Contributions of the Advanced Practice Nurse in the chronicity. Learning from experiences. Enferm. Clín..

[19] Servicio Andaluz de Salud (2003). Guía de procedimientos. Rehabilitación y Fisioterapia en Atención Primaria.

[20] Ollero Baturone M., Bernabeu-Wittel M., Espinosa Almendro J.M. (2007). Atención a Pacientes Pluripatológicos: Proceso Asistencial Integrado.

[21] Smith S.M., Wallace E., O’Dowd T., Fortin M. (2016). Interventions for improving outcomes in patients with multimorbidity in primary care and community settings. Cochrane Database Syst. Rev..

[22] Shah S., Vanclay F., Cooper B. (1989). Improving the sensitivity of the Barthel index for stroke rehabilitation. J. Clin. Epidemiol..

[23] World Health Organizations (2011). Informe Mundial Sobre la Discapacidad. http://www.who.int/iris/bitstream/10665/75356/1/9789240688230_spa.pdf.

[24] Spanish Institute of Statistics., Ministerio de Sanidad y Política Social., Unidad de Pacientes Pluripatológicos (2009). Estándares y Recomendaciones.

[25] Nieto M.D. (2007). IMPACTO: Implantación del Plan de Asistencia Continuada a Pacientes Pluripatológicos. Impacto Sobre la Evolución Natural de la Enfermedad, el Deterioro Funcional y la Calidad de Vida. http://www.fesemi.org/documentos/grupos/edad−avanzada/publicaciones/anexo−impacto.pdf.

[26] Fernández R., Ramos M.R., Gallardo J.S., Navarro Torrente M.D., López Montoya I.A. (2008). Ibáñez Gil E. Efecto de la intervención de la Enfermera Hospitalarias de Enlace en la calidad de vida y estado funcional en pacientes crónicos, frágiles, pluripatológicos, y sus cuidadoras. Biblioteca LasCasas.

[27] Ramírez-Duque N., Ollero-Baturone M., Bernabeu-Wittel M., Rincón-Gómez M., Ortiz-Camuñez M.A., García-Morillo S. (2008). Características clínicas, funcionales, mentales y sociales de pacientes pluripatológicos. Estudio prospectivo durante un año en Atención Primaria. Rev. Clín. Española.

[28] García-Fernández F.P., Carrascosa-García M.I. (2008). Resultados de las intervenciones enfermeras en el proceso pluripatológico para mejorar la práctica clínica. Gerokomos.

[29] Zambrana García J.L., Velasco Malagón M.J., Díez García F., Cruz Caparrós G., Martín Escalante M.D., Adarraga Cansino M.D. (2005). Características clínicas diferenciales de los enfermos pluripatológicos hospitalizados en servicios de Medicina Interna. Rev. Clín. Española.

[30] Bertel−De la Hoz A.M. (2012). Riesgo a enfermar y sobrecarga del cuidador principal del anciano dependiente. Revista de Ciencias Biomédicas.

[31] Da Silva M.A., Braga Marques M., Da Silva Bruno C.T. (2009). Evaluación de la Presencia del Síndrome de Burnout en Cuidadores de Ancianos. Enferm. Glob..

[32] De Valle-Alonso M.J., Hernández-López I.E., Zúñiga-Vargas M.L., Martínez-Aguilera P. (2015). Sobrecarga y Burnout en cuidadores informales del adulto mayor. Enferm. Univ..

[33] Domínguez-Sosa G., Zavala-González M.A., De la Cruz-Méndez D.C., Ramírez-Ramírez M.O. (2010). Síndrome de sobrecarga en cuidadores primarios de adultos mayores de Cárdenas, Tabasco, México. Rev. MED UIS.

[34] Giraldo C.I., Franco G.M., Correa L.S., Salazar M.O., Tamayo A.M. (2005). Cuidadores familiares de ancianos: Quiénes son y cómo asumen este rol. Rev. Fac. Nac. Salud Pública.

[35] Ocampo M.J., Herrera J.A., Torres P., Rodríguez Matiz J.H., Loboa L., García C.A. (2007). Sobrecarga asociada con el cuidado de ancianos dependientes. Rev. Colomb. Med..

[36] Delgado Ojeda M.A. (2000). Rehabilitación y Fisioterapia en Geriatría.

[37] García-Morillo J.S., Bernabeu-Wittel M., Ollero-Baturone M., Aguilar-Guisad M., Ramírez-Duque N., González M.A., Limpo P., Romero-Carmona S., Cuello-Contreras J.A. (2005). Incidencia y características clínicas de los pacientes con pluripatología ingresados en una unidad de medicina interna. Med. Clín..

[38] Vega-Ramírez F.A., López-Liria R., Granados-Gámez G., Aguilar-Parra J.M., Padilla-Góngora D. (2017). Analysis of home-based rehabilitation in patients with motor impairment in primary care: A prospective observational study. BMC Geriatr..

[39] Melguizo M. (2011). De la enfermedad crónica al paciente en situación de cronicidad. Atención Primaria.

[40] Sánchez-Martín C.I. (2014). Chronic diseases and complexity: New roles in nursing. Advanced practice nurses and chronic patient. Enferm. Clín..

[41] López-Liria R., Vega-Ramírez F.A., Rocamora-Pérez P., Aguilar-Parra J.M., Padilla-Góngora D. (2016). Comparison of Two Post-Stroke Rehabilitation Programs: A Follow-Up Study among Primary versus Specialized Health Care. PLoS ONE.

[42] López-Liria R., Padilla-Góngora D., Catalán-Matamoros D., Rocamora-Pérez P., Pérez-de la Cruz S., Fernández-Sánchez M. (2015). Home-based versus hospital based rehabilitation program after Total knee replacement. BioMed Res. Int..

[43] Orueta J.F., Mateos del Pino M., Barrio I., Nuño R., Cuadrado M., Sola C. (2012). Estratificación dela población en el País Vasco: Resultados en el primer año de implantación. Atención Primaria.

